# Photovoltaic panels have altered grassland plant biodiversity and soil microbial diversity

**DOI:** 10.3389/fmicb.2022.1065899

**Published:** 2022-12-15

**Authors:** Zhenyin Bai, Aomei Jia, Zhenjian Bai, Shanmin Qu, Meng Zhang, Linghang Kong, Renhao Sun, Mingjun Wang

**Affiliations:** ^1^College of Animal Science and Technology, Northeast Agricultural University, Harbin, China; ^2^College of Animal Science and Veterinary Medicine, Heilongjiang Bayi Agricultural University, Daqing, China

**Keywords:** photovoltaic panel, grassland, aboveground biomass, plant community composition, soil microbial diversity

## Abstract

**Introduction:**

Human concerns about fossil fuel depletion, energy security and environmental degradation have driven the rapid development of solar photovoltaic (PV) power generation. Most of the photovoltaic power generation plants are concentrated in desert, grassland and arable land, which means the change of land use type. However, there is still a gap in the research of the PV panel layout on grassland plant species diversity and ecological function.

**Methods:**

In this study, Illumina high-throughput sequencing technology was used to investigate the effects of PV panel arrangement on grassland plant species diversity and soil microbial diversity. In view of the differences in the microclimate at different sites of the PV panels, quadrates were arranged in front edge (FE), beneath the center of each panel (BP), back edge (BE), the uncovered interspace adjacent to each panel (IS) and the undisturbed grassland around the PV panels (Control), respectively.

**Results:**

PV panels (especially FE) significantly increased the total aboveground productivity (total AGB) and plant species diversity in grasslands. FE increased precipitation accumulation and plant species diversity directly and indirectly changed the diversity of soil bacterial and fungal communities. PV panels decreased the relative abundance of Actinobacteriota, while increased the relative abundance of Proteobacteria, Acidobacteriota, and Methylomirabilota. EC, Margalef’ s richness and total AGB were the main factors affecting the composition of bacterial communities, while alkaline hydrolysis nitrogen (AN) and available phosphorus (AP) were the main factors affecting the composition of fungal communities.

**Discussion:**

In conclusion, the arrangement of PV panels increased the plant species diversity and soil microorganisms in grassland. This study provides important information for further understanding the impact of PV panels on grassland ecosystem function and is of great significance for maintaining grassland ecosystem function.

## Introduction

Human concerns over fossil fuel depletion, energy security and environmental degradation have led to an increasing demand for clean renewable energy (Ding et al., 2016). The two outstanding characteristics of zero pollution and zero emissions make solar photovoltaic power (PV) a better energy source and an ideal alternative to traditional fossil fuels (Joshi and Arora, 2017). By the end of 2019, the global total installed capacity of PV had reached 505 GW (REN21, 2019), and it is still growing rapidly. PV generation has become an important pillar of national energy development strategies (Wu et al., 2021). However, PV is spatially intensive, large-scale and non-integrated, and PV deployment is estimated to require more land area than coal nuclear or natural gas technologies (Fthenakis and Kim, 2009). At present, the construction of PV plants is mostly concentrated in desert, grassland and arable land, which means the change of land use types (Mac Kay, 2013). Grassland is the world’s largest terrestrial ecosystem and an important part of the global natural ecosystem. They play an important role in the development of animal husbandry, windbreak and sand control, soil and water conservation, biodiversity protection and ecological balance (Li et al., 2006; Wen et al., 2013). Although PV generation has many social functions, such as helping to achieve low emission rate, alleviating energy crisis, improving the efficiency of resource utilization, there is still a gap in the research of PV panel layout on grassland diversity and ecological function.

The physical presence of PV panels will affect solar radiation flux (temperature), wind speed and turbulence (potential evaporation) and precipitation distribution under PV panels (Armstrong et al., 2014). A large number of studies have shown that PV panels reduce the amount of solar radiation received by the local surface and the atmospheric temperature in the growing season (from April to August) by converting part of the solar energy into electric energy (Armstrong et al., 2014; Liu et al., 2019; Yue et al., 2021). The shielding effect of PV panels leads to uneven precipitation distribution (Elamri et al., 2018; Li Y. et al., 2018), the presence of PV panels can concentrate water at its lower edge, which increases the local heterogeneity of soil water distribution and creates more permanent water storage under PV panels (Adeh et al., 2018; Yue et al., 2021). In addition, the lower potential evaporation caused by the reduction of solar radiation directly below the PV panels will lead to a reduction in drought, an increase in soil water availability, and help reduce water loss in arid climates (Marrou et al., 2013; Adeh et al., 2018; Choi et al., 2020).

The linkage changes of species interactions under climate change affect the biodiversity and function of terrestrial ecosystems (Walther et al., 2002; Gottfried et al., 2012; Langley and Hungate, 2014; Rudgers et al., 2014; Crowther et al., 2016). Given that light, temperature and water are the three key factors for plant growth and ecosystem function (Martens et al., 2000; Dupraz et al., 2011; Li W. H. et al., 2018), individual plant species differ in their ability to feedback climate change or survive under changing conditions through adaptation or plasticity (Fernandez-Going et al., 2013; Shi et al., 2015). The interaction between local microclimate changes caused by PV panels may affect plant community structure (Cleland et al., 2004; Adler et al., 2006; Yang et al., 2011), and directly/indirectly affect microbial community structure through changes in the plant community composition (Ren et al., 2018; Toju et al., 2018; Shi et al., 2020). Therefore, it is necessary to better understand the human interference (i.e., the laid of pv panels), climate change and bio-abiotic interactions affect how grassland ecosystem structure and function. This will provide a theoretical reference for the selection and construction of scientific, reasonable and efficient PV power plants, and provide scientific basis for vegetation and soil restoration after PV power plants construction.

Grassland ecosystems are considered to be one of the most sensitive ecosystems to climate change (Piao et al., 2012; Chen et al., 2013). Microclimate change caused by human disturbance will have a profound impact on grassland ecosystem function. Therefore, understanding the impact of PV panels on grassland ecosystem is of great significance for maintaining grassland ecosystem function. In this study, the PV power plant is located in Datong District, Daqing City. In the past, many large areas of grassland were constructed as PV power stations. The objectives of this study were: (1) to investigate the effects of PV panels laying on grassland productivity, plant community composition and species diversity, soil properties, microbial community composition and diversity; (2) to clarify the intrinsic relationship between plant species diversity, soil properties and soil microbial changes.

## Materials and methods

### Study site

The study area is located in the western Songnen Plain, which belongs to Datong District of Daqing City (46°10 ‘11’ N, 124°53 ‘56’ E; 135 m asl), which has a mid-temperate continental monsoon climate. Light is sufficient, precipitation is less, wind and drought in the same period, rain and heat in the same season. The mean annual temperature is 4.2°C, the mean temperature of the coldest month is-18.5°C, and the extreme minimum temperature is-39.2°C. The average temperature of the hottest month is 23.3°C, and the extreme maximum temperature is 39.8°C. The average annual precipitation is 400-500 mm, and the precipitation period is mostly concentrated in June to September. The average annual frost-free period is 143 days. The average annual wind speed is 3.8 m/s. The annual evaporation is 1,635 mm, the annual dryness is 1.2, the annual sunshine duration is 2,726 h, and the annual total solar radiation is 491.4 kJ/cm^2^. The main soil types were meadow and marsh soil and the grassland type in the study area is warm meadow grassland, and the main plant types are *Puccinellia tenuiflora* (Griseb.) Scribn. et Merr., *Phragmites australis* (Cav.) Trin. ex Steud. and *Suaeda glauca* (Bunge) Bunge.

### Experiment design

**Figure 1:**
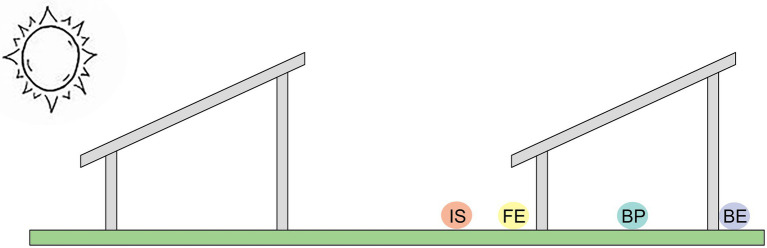
Different sites under the PV panels (FE: front edge of each panel, BP: beneath the center of each panel; BE: back edge of each panel; IS: the uncovered interspace adjacent to each panel; Control: the undisturbed grassland around the PV panels).

The PV power station was randomly divided into three areas, and three PV panels were randomly selected in each area, making a total of nine PV panels as the research object. In addition, an undisturbed grassland around each area was selected as control, and a total of three control grasslands were selected, with a spacing of 20 m between the control grassland and the study areas. In view of the differences in the microclimate at different sites of the PV panel, quadrates were arranged in front edge (FE), beneath the center of each panel (BP), back edge (BE), the uncovered interspace adjacent to each panel (IS) and the undisturbed grassland around the PV panels (Control), respectively (Figure 1). The size of each quadrat is 1 × 1 m, four quadrats are arranged under each PV panel, and one quadrat is arranged in each control area, a total of 39 quadrats.

### Plant sampling and analysis

During the study, all plant data were measured at the end of July, when the plants were in full bloom. The plant community was divided into four functional groups:PG (perennial grasses); PS (perennial sedges); PF (perennial forbs); ABH (annual and biennial herbs). After litter removal, we measured total plant community coverage, height, coverage and density of each species, and density of each plant functional group within each quadrat. The height of each plant functional group was obtained by calculating the mean of plants within the group. Each plant was then trimmed to floor level and placed in marked paper bags by species. Then it was dried at 65°C for 48 h and weighed to obtain the aboveground biomass (AGB) of each species, and AGB of each plant functional group and total AGB were calculated. Plant Margalef’s index, Shannon-Wiener index, Simpson index were used to illustrate biodiversity in this study. Margalef’s richness index was calculated as follows:


$$R_{1}=\frac{S-1}{InN}$$

where N is the total number of individuals of all species, and S is the number of species. Shnnon-Wiener index was calculating as follows:


$$H^{\prime }=-\sum P_{i}InP_{i}$$

where Pi is the proportion of individual species i representing the relative density of plant species (species density/total density for all species × 100). Simpson diversity index was calculated as follows:


$$D=1-\sum {\left(P_{i}\right)}^{2}$$

### Soil Sampling and Analysis

After measuring the AGB parameters, soil was sampled at five points with a soil coring machine. Soil samples were collected at a depth of 0–20 cm. Five samples from the same quadrat at the same depth were thoroughly mixed to remove root and gravels, quickly packed into sterile sealable bags, placed in ice boxes for refrigeration, and promptly shipped to the laboratory. The sample was divided into two parts. Part of the soil was air-dried, ground and sieved to determine the physical and chemical properties of the soil. The remaining parts were stored at-80°C for soil microbial analysis.

### Soil nutrient determination

Soil pH and electrical conductivity (EC; an important indicator of salinity) were determined with soil (air-dried)/water (1:5, w/v) suspensions (Bao, 2008), and soil organic matter (SOM) was assessed by K_2_CrO_7_ volumetric method (External heating method) (Sparks et al., 1996). Total nitrogen (TN) and alkali-hydrolyzed nitrogen (AN) were measured by Kjeldahl method and alkali-hydrolyzed diffusion method (Lu, 1999). Phosphorus (P) was extracted by HClO_4_-H2SO_4_ method, then added the molybdenum-antimony anti-reagent, colorimetric was performed at 880 nm or 700 nm wavelength, and calculated the total phosphorus (TP) content (Bao, 2008). Available phosphorus (AP) was extracted with 0.5 M NaHCO_3_ (pH 8.5), and was determined using molybdenum blue method (UV-752 Shanghai, China; Bao, 2008). Determination of total potassium and available potassium by flame photometry (Bao, 2008).

### Soil DNA extraction, illumina sequencing, and data analysis

Total DNA was extracted from 0.25 g of Soil using the FastDNA® Spin Kit for Soil (MP Biomedicals, USA) isolation kit according to the manufacturer’s requirements. After DNA extraction, the extracted genomic DNA was detected by 1% agarose gel electrophoresis. For MiSeq library construction, bacterial V3 and V4 hypervariable regions were amplified using primers 338F (5′ -ACTCCTACGGGAGGCAGCA-3′) and 806R (5′ -GGACTACHVGGGTWTCTAAT-3′). Primer ITS1F was used (5’-CTTGGTCATTTAGAGGAAGTAA-3′) and ITS2 (5’-GCTGCGTTCTTCATCGATGC-3′) were used to amplify the hypervariable region of fungal ITS1. Specific primers with barcode were synthesized according to the specified sequencing region. The amplification process was pre-denaturation at 95°C for 5 min, 25 cycles of 95°C for 30 s, 50°C annealing for 30 s, 72°C for 40 s, and 72°C for 7 min of extension. PCR products of the same sample were mixed and detected by 2% agarose gel electrophoresis. PCR products were recovered by gel cutting using AxyPrepDNA gel recovery kit (AXYGEN Company) and eluted with Tris_HCl. Electrophoresis was performed on 2% agarose. PCR products were quantified with the QuantiFluor™ -ST Blue fluorescence Quantification System (Promega). The PCR products were purified with TruSeqTM DNA Sample Prep Kit to construct the Miseq library. Illumina Miseq platform was used for on-machine sequencing of the constructed library.

The reads of each sample were spliced using FLASH (version 1.2.11) software (Magoc and Salzberg, 2011), primers were removed, and sequences were trimmed to remove low-quality sequences. Sequences were clustered at 97% similarity level (OTU) and screened using USEARCH (version 10.0) with a threshold of 0.005% for all sequences (Edgar, 2013). The RDP software (Version 2.2) was then used for comparison with the Silva and UNITE databases, with confidence intervals of 80% for bacterial and fungal classification (Wang et al., 2007; Quast et al., 2013). Mothur software was used to calculate Chao1, Simpson diversity, Shannon’s evenness, and Good’s coverage to estimate bacterial and fungal diversity and richness.

### Statistical analysis

All statistical analyses were performed using R (4.1.2) software. One-way analysis of variance (ANOVA) and TukeyHSD test in “Agricolae” package were used to analyze the significant differences between groups in plant diversity, soil properties, soil bacterial α diversity and relative abundance of dominant phyla, and ANOVA was used to test the effects of PV panels on plants, soil, bacteria and fungi. Spearman rank correlation coefficient was calculated to analyze the correlation between vegetation, soil properties and soil microorganisms. Based on Bray-Curtis distance matrix, the similarity analysis (ANOSIM) of 999 permutations was performed, and the PCoA visualization of plant community composition at different sites was performed. Based on Bray-Curtis distance matrix, NMDS was used to visualize the composition of bacterial and fungal communities at different sites. Linear discriminant analysis (LDA) effect size (LEfSe) method was used to identify significantly different characteristics of bacteria and fungi at different sites by biomarkers, and the impact size of each feature was assessed with a threshold of 3 and a significant α of 0.5. The “vegdist” package was used to conduct RDA analysis of plant diversity, soil properties and bacterial and fungal communities, and 999 permutation similarity analysis (ANOSIM) was performed to infer potential relationships between microbial community composition and measured plant diversity and soil properties. SEM models were constructed using Amos 26 (AMOS IBM, USA) to assess the direct and indirect effects of vegetation and soil properties on bacterial and fungal community diversity.

## Results

### Effects of PV panels on plant community and soil properties

PV panels had significant effects including the Margalef’s richness index, Shnnon-Wiener index and Simpson diversity index of the plants. From FE to IS, BP, BE and Control, most diversity indices decreased (*p* < 0.05). For different sites under the PV panel, the diversity of FE plants increased the most significantly, while the diversity of BE plants increased the least. From the perspective of homogeneity, compared with IS, most diversity indices increased in FE and decreased in BP and BP (Table 1; Supplementary Figure S1). PV panels had significant effects on total AGB and community total coverage (*p* < 0.05) (Table 2), and total AGB decreased from FE to BE, IS, BP, and Control. Compared with Control, total AGB under FE, BE, IS, and BP increased by 80.02, 52.01, 43.04, and 17.10%, respectively (Table 2; Supplementary Figure S2). PV panels significantly increased the total coverage of plant communities, which decreased from FE to BP, IS, BE and Control. Compared with Control, the total plant community coverage under FE increased by 80.02% (Table 2). Compared with IS, total AGB and total coverage increased significantly under FE and decreased significantly under BP, but there was no significant difference under BE. Compared with Control, the total coverage of plant communities increased most significantly under FE, increasing by 80.02%. However, the total coverage of plant communities increased the least under BP, with an increase of 17.10% (Table 2).

**Table 1:** One-way ANOVA analysis of the effects of PV panels on plant species, soil bacterial, and fungal diversity (FE: front edge of each panel, BP: beneath the center of each panel; BE: back edge of each panel; IS: the uncovered interspace adjacent to each panel; Control: the undisturbed grassland around the PV panels).

		Site					F	P
		Control	IS	FE	BP	BE		
Plant α diversity	Margalef’s richness	0.43 ± 0.08 b	0.95 ± 0.21 ab	1.04 ± 0.35 a	0.72 ± 0.33 ab	0.71 ± 0.34 ab	3.343	0.021
	Shnnon-Wiener	0.56 ± 0.34 c	1.32 ± 0.27 ab	1.46 ± 0.53 a	0.96 ± 0.35 abc	0.87 ± 0.38 bc	5.207	0.002
	Simpson diversity	0.31 ± 0.23 c	0.66 ± 0.07 ab	0.70 ± 0.18 a	0.49 ± 0.17 abc	0.43 ± 0.24 bc	4.925	0.003
Bacteria α diversity	Chao1 richness	2866.99 ± 150.05 c	4023.72 ± 280.48 ab	4680.33 ± 369.26 a	3595.27 ± 714.17 bc	3776.64 ± 670.66 bc	8.781	<0.001
	Simpson diversity	0.03 ± 0.00 a	0.01 ± 0.01 b	0.01 ± 0.00 b	0.01 ± 0.01 b	0.01 ± 0.01 b	6.368	<0.001
	Shannon’s evenness	4.87 ± 0.08 c	6.05 ± 0.43 ab	6.43 ± 0.43 a	5.75 ± 0.51 b	5.98 ± 0.47 ab	7.474	<0.001
	Good’s coverage	0.99 ± 0.00 a	0.99 ± 0.00 ab	0.98 ± 0.00 b	0.99 ± 0.01 ab	0.98 ± 0.00 ab	2.730	0.045
Fungi α diversity	Chao1 richness	170.43 ± 36.09 b	290.54 ± 87.39 ab	447.78 ± 176.34 a	292.31 ± 163.00 ab	318.92 ± 115.74 ab	3.065	0.029
	Simpson diversity	3.09 ± 0.52 a	3.15 ± 0.67 a	3.35 ± 0.62 a	3.22 ± 0.61 a	2.93 ± 0.41 a	0.425	0.789
	Shannon’s evenness	0.14 ± 0.12 a	0.13 ± 0.09 a	0.13 ± 0.08 a	0.11 ± 0.06 a	0.17 ± 0.11 a	0.591	0.672
	Good’s coverage	1.00 ± 0.00 a	1.00 ± 0.00 a	1.00 ± 0.00 a	1.00 ± 0.00 a	1.00 ± 0.00 a	2.228	0.087

**Table 2:** One-way ANOVA analysis of the effects of PV panels on plant communities and functional groups (PG: perennial grasses; PS: perennial sedges; PF: perennial forbs; ABH: annual and biennial herbs).

	Control	IS	FE	BP	BE	*F*	*P*
Total AGB	312.32 ± 53.99b	446.74 ± 168.52ab	565.24 ± 102.21a	365.73 ± 130.26b	474.77 ± 171.52ab	3.039	0.030
Total coverage	50.00 ± 5.00b	66.11 ± 9.28ab	78.11 ± 7.04a	68.33 ± 11.73ab	65.00 ± 10.61b	5.437	<0.001
PG coverage	46.33 ± 5.51a	42.61 ± 16.96a	47.62 ± 25.17a	41.00 ± 22.06a	38.11 ± 20.51a	0.274	0.893
PF coverage	0.00 ± 0.00a	3.00 ± 6.78a	11.62 ± 13.13a	5.89 ± 14.30a	0.06 ± 0.17a	1.834	0.145
PS coverage	0.00 ± 0.00a	13.39 ± 13.93a	12.01 ± 14.56a	1.00 ± 1.80a	7.67 ± 11.30a	2.057	0.108
ABH coverage	5.33 ± 3.06a	19.39 ± 17.49a	34.14 ± 16.01a	24.89 ± 24.84a	26.96 ± 23.66a	1.360	0.268
PG height	219.92 ± 194.42a	70.50 ± 15.37b	65.91 ± 17.74b	50.31 ± 18.67b	65.39 ± 11.92b	7.114	<0.001
PF height	0.00 ± 0.00b	6.67 ± 15.21ab	21.00 ± 20.83a	3.07 ± 6.13ab	2.22 ± 6.67b	3.187	0.025
PS height	0.00 ± 0.00b	44.00 ± 23.69a	29.39 ± 24.80ab	9.44 ± 17.40b	39.78 ± 23.77a	4.687	0.004
ABH height	37.00 ± 24.64a	28.88 ± 16.96a	35.26 ± 8.74a	25.51 ± 12.44a	43.47 ± 17.84a	1.855	0.141
PG abundance	275.33 ± 200.14a	178.89 ± 198.77a	180.89 ± 119.44a	149.00 ± 149.12a	118.22 ± 132.35a	0.643	0.636
PF abundance	0.00 ± 0.00a	10.11 ± 29.96a	30.33 ± 38.80a	13.00 ± 26.23a	0.33 ± 1.00a	1.621	0.192
PSabundance	0.00 ± 0.00a	30.11 ± 29.54a	28.11 ± 28.79a	4.89 ± 10.34a	28.22 ± 29.45a	2.117	0.100
ABH abundance	24.00 ± 23.30a	124.01 ± 147.08a	239.11 ± 210.53a	249.22 ± 261.02a	195.89 ± 212.45a	1.039	0.402
PG frequency	1.31 ± 0.00a	1.60 ± 0.24a	1.54 ± 0.35a	1.27 ± 0.38a	1.28 ± 0.46a	1.635	0.188
PF frequency	0.00 ± 0.00a	0.03 ± 0.06a	0.20 ± 0.20a	0.09 ± 0.18a	0.02 ± 0.06a	2.879	0.037
PS frequency	0.00 ± 0.00a	0.55 ± 0.22a	0.43 ± 0.33ab	0.20 ± 0.30ab	0.41 ± 0.28ab	3.444	0.018
ABH frequency	0.41 ± 0.27a	1.18 ± 0.57a	1.06 ± 0.61a	0.94 ± 0.61a	1.01 ± 0.37a	1.211	0.324
PG AGB	288.50 ± 54.22a	207.13 ± 114.39a	241.59 ± 100.44a	165.19 ± 81.09a	183.98 ± 107.06a	1.296	0.291
PF AGB	0.00 ± 0.00a	14.06 ± 41.69a	24.45 ± 29.10a	8.85 ± 20.67a	0.32 ± 0.95a	1.109	0.368
PS AGB	0.00 ± 0.00b	77.87 ± 81.59a	39.75 ± 56.85ab	1.55 ± 2.81b	31.99 ± 56.86ab	2.474	0.062
ABH AGB	23.82 ± 19.87a	137.42 ± 153.47a	222.04 ± 180.98a	190.14 ± 201.34a	258.48 ± 276.91a	0.973	0.435

**Figure 2:**
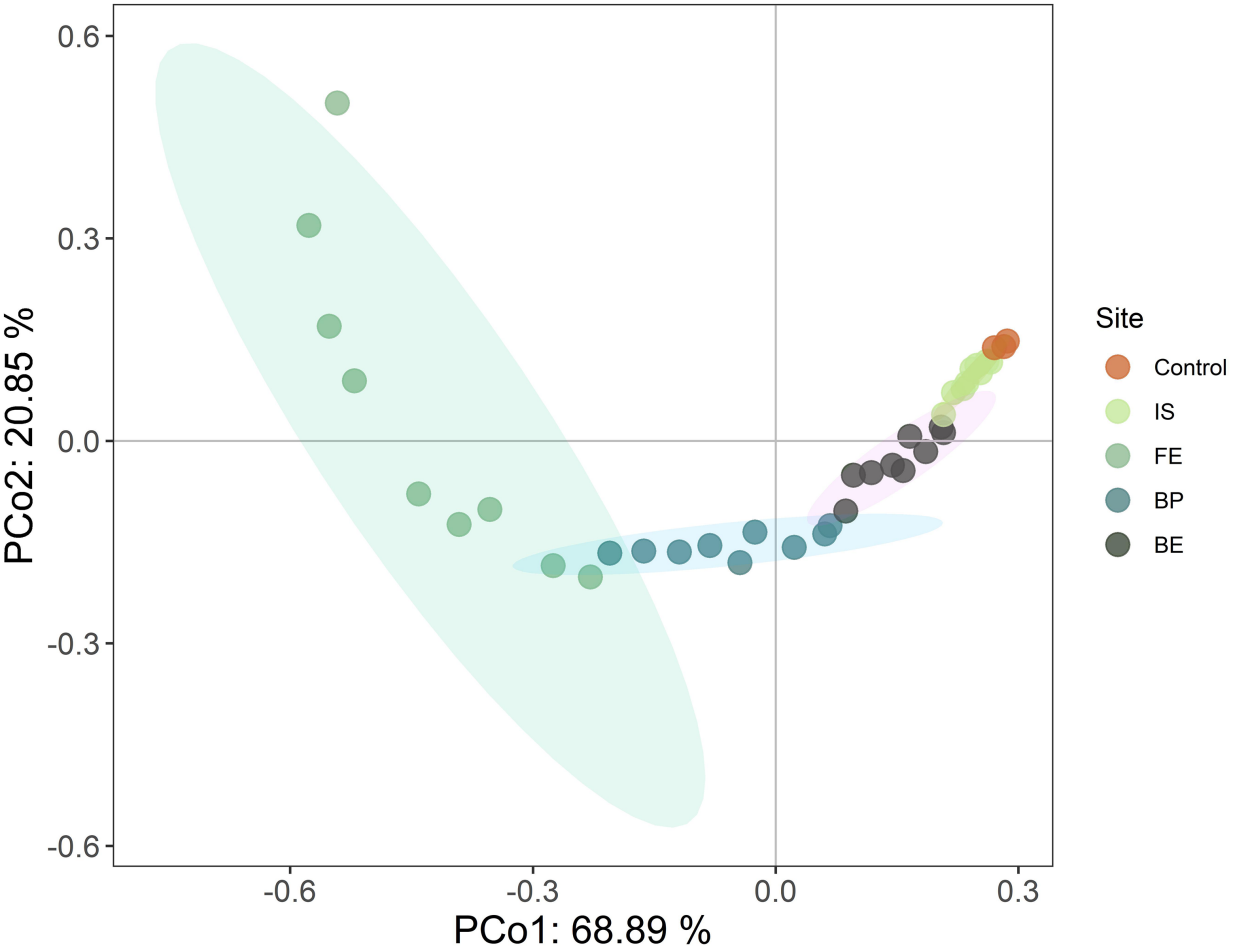
Principal coordinate analysis (PCoA) of plant community composition at different sites of PV panels.

The results of PCoA based on Bray-Crutis showed that PCo1 and PCo2 explained 68.89 and 20.85% of the variation in plant community composition, respectively. The plant community composition was significantly separated between Control and PV panels, indicating that PV panels changed the plant community composition, and the plant composition at different sites under PV panels was significantly different (*F* = 26.235; *p* < 0.001). PV panels promoted the growth of PF, PS and ABH, while inhibited the growth of PG (*R*^2^ = 0.755, *p* = 0.001) (Figure 2; Table 2). PV panels had significant effects on the height and frequency of plant functional groups (*p* < 0.05). However, there were significant differences in different sites under the PV panels. The PF height under FE increased the most significantly, but there was no significant difference between BE and Control. The PS height increased most significantly under IS and BE, but there was no significant difference between Control under BP. There was no significant difference in PG height between different sites of PV panels. There was no significant difference in the coverage and richness of each functional group at different sites under the PV panels compared with the Control. FE, BP and BE decreased the frequency of PS, but there was no significant difference between IS and Control. The PV panel significantly increased PS AGB, and IS was the most significant, while BP was the least (Table 2).

Except for TP, PV panel had no significant effect on soil properties (*p* > 0.05). Although there was no statistical difference in soil properties between different sites under PV panels and Control. However, compared with the Control, the pH, EC, TK and AK contents at different sites under the PV panel were slightly decreased, while AN, TP and AP were slightly increased. For different sites under the PV panel, most soil properties were highest under BP (Table 3).

**Table 3:** One-way ANOVA analysis of the effects of PV panels on soil properties.

Site	pH	EC (mS/cm)	SOC (g/kg)	TN (g/kg)	AN (mg/kg)	TP (g/kg)	AP (mg/kg)	TK (g/kg)	AK (mg/kg)
Control	8.90 ± 0.04a	3.2 ± 1.24a	10.06 ± 0.46a	1.23 ± 0.47a	150.07 ± 28.12a	0.95 ± 0.38a	175.52 ± 29.66a	36.75 ± 1.24a	144.26 ± 1.44a
IS	8.59 ± 0.41a	1.84 ± 0.65a	9.98 ± 2.12a	1.09 ± 0.36a	159.72 ± 80.16a	1.00 ± 0.23a	197.89 ± 54.65a	36.66 ± 1.49a	142.97 ± 3.41a
FE	8.54 ± 0.41a	1.94 ± 0.95a	11.10 ± 1.51a	1.56 ± 0.56a	156.29 ± 49.67a	1.29 ± 0.46a	189.22 ± 42.50a	36.10 ± 1.86a	143.21 ± 2.70a
BP	8.59 ± 0.25a	1.95 ± 1.13a	11.15 ± 1.74a	1.22 ± 0.43a	193.34 ± 94.41a	1.48 ± 0.41a	185.51 ± 68.83a	35.41 ± 2.194a	141.62 ± 3.33a
BE	8.62 ± 0.49a	1.69 ± 0.65a	10.89 ± 1.36a	1.52 ± 0.72a	183.17 ± 69.24a	1.39 ± 0.23a	190.99 ± 72.62a	36.60 ± 1.15a	140.31 ± 2.64a
*F*	0.519	1.696	0.859	1.279	0.477	2.973	0.097	0.858	1.769
*P*	0.722	0.174	0.498	0.298	0.752	0.033*	0.983	0.499	0.158

### Effects of PV panels on soil microbial community diversity and composition

A total of 2,751,094 and 4,879,890 effective bacterial and fungal sequences were obtained from 39 samples after Illumina high-throughput sequencing and filtering to remove low-quality sequences. The effective sequence length of bacteria was mainly distributed in the range of 400-440 bp, and that of fungi was mainly distributed in the range of 220-240 bp. There were 23,143 bacterial OTUs and 3,064 fungal OTUs in all samples. The bacterial OTUs of each sample ranged from 43,933 to 119,026, with an average of 70,541, and the fungal OTUs ranged from 104,110 to 148,006, with an average of 125,125, and the homology threshold was 97%. The Good’s coverage of bacteria and fungi of all samples were higher than 98.61 ± 0.48% and 99.97 ± 0.01%, respectively, indicating that the sequencing quality was good.

PV panels had significant effects including bacterial Chao1 richness, Simpson diversity, Shannon’s evenness and Good’s coverage, and fungi Chao1 richness (*p* < 0.05) (Table 1). As for bacteria, the Chao1 richness and Shannon’s evenness of bacteria increased by PV panels, including the most significant increase in FE, but the least increase in BP, and no significant difference between IS and BE. The Simpson diversity of bacteria was decreased by PV panels, and there was no significant difference among different sites of PV panels (*p* > 0.05). For fungi, Chao1 richness was significantly improved by PV panels, including FE which was the most significant, but there was no significant difference among IS, BP, and BE. The fungi Simpson diversity and Shannon’s evenness were not significantly affected by PV panels (Table 1; Supplementary Figure S3). Spearman rank correlation analysis showed that bacteria Chao1 richness was positively correlated with Margalef’s richness, Shnnon-Wiener and Simpson diversity. Shannon’s evenness was positively correlated with Margalef’s richness, Shnnon-Wiener and total AGB, and negatively correlated with EC. Simpson diversity was negatively correlated with Margalef’s richness, Shnnon-Wiener and total AGB, and positively correlated with EC. Fungi Chao1 richness was positively correlated with AP, Shnnon-Wiener and Simpson diversity, but negatively correlated with EC (Table 4).

**Table 4:** Spearman rank correlation analysis of soil bacteria and fungi with plant species diversity and soil properties.

		TN	TP	AN	AP	PH	EC	SOC	TK	AK	Margalef	Shnnon	Simpson	Total AGB
Bacteria	Chao1	0.272	0.037	−0.073	0.124	−0.266	−0.235	0.053	−0.061	−0.046	0.545	0.614	0.646	0.183
	Shannon	0.076	0.056	0.083	0.105	−0.189	−0.416	0.102	−0.026	−0.060	0.367	0.481	0.546	0.346
	Simpson	−0.069	0.016	−0.029	−0.012	0.186	0.390	−0.045	0.032	0.024	−0.379	−0.525	−0.579	−0.356
	Good’s Coverage	−0.126	−0.118	−0.124	−0.134	0.221	0.291	−0.151	0.018	0.148	−0.241	−0.384	−0.384	−0.251
Fungi	Chao1	−0.045	0.249	0.178	0.372	−0.154	−0.338	0.226	0.031	−0.099	0.243	0.320	0.317	0.120
	Shannon	−0.119	−0.140	0.076	0.195	−0.061	0.113	0.062	0.043	−0.155	0.212	0.160	0.036	−0.192
	Simpson	0.151	0.267	0.003	−0.074	0.013	−0.109	0.068	−0.124	0.142	−0.149	−0.165	−0.076	0.193
	Good’s Coverage	0.076	−0.103	−0.145	−0.423	−0.018	0.124	−0.046	−0.308	−0.043	−0.063	−0.187	−0.196	−0.021

Based on Bray-Curtis distance matrix, we performed NMDS visualization for bacteria and fungi in different sites. Bacterial and fungal Stress were 0.0906 and 0.1673, respectively. Permanova analysis results showed that the bacterial community composition was significantly different among different sites (*R*^2^ = 1.564, *p* = 0.030), while the fungal community composition was similar among different sites (R^2^ = 1.020, *p* = 0.409). However, PV panels did not significantly affect the bacterial and fungal community composition, and bacterial and fungal community composition was similar between Control and different PV panel sites (Supplementary Figure S4).

**Figure 3:**
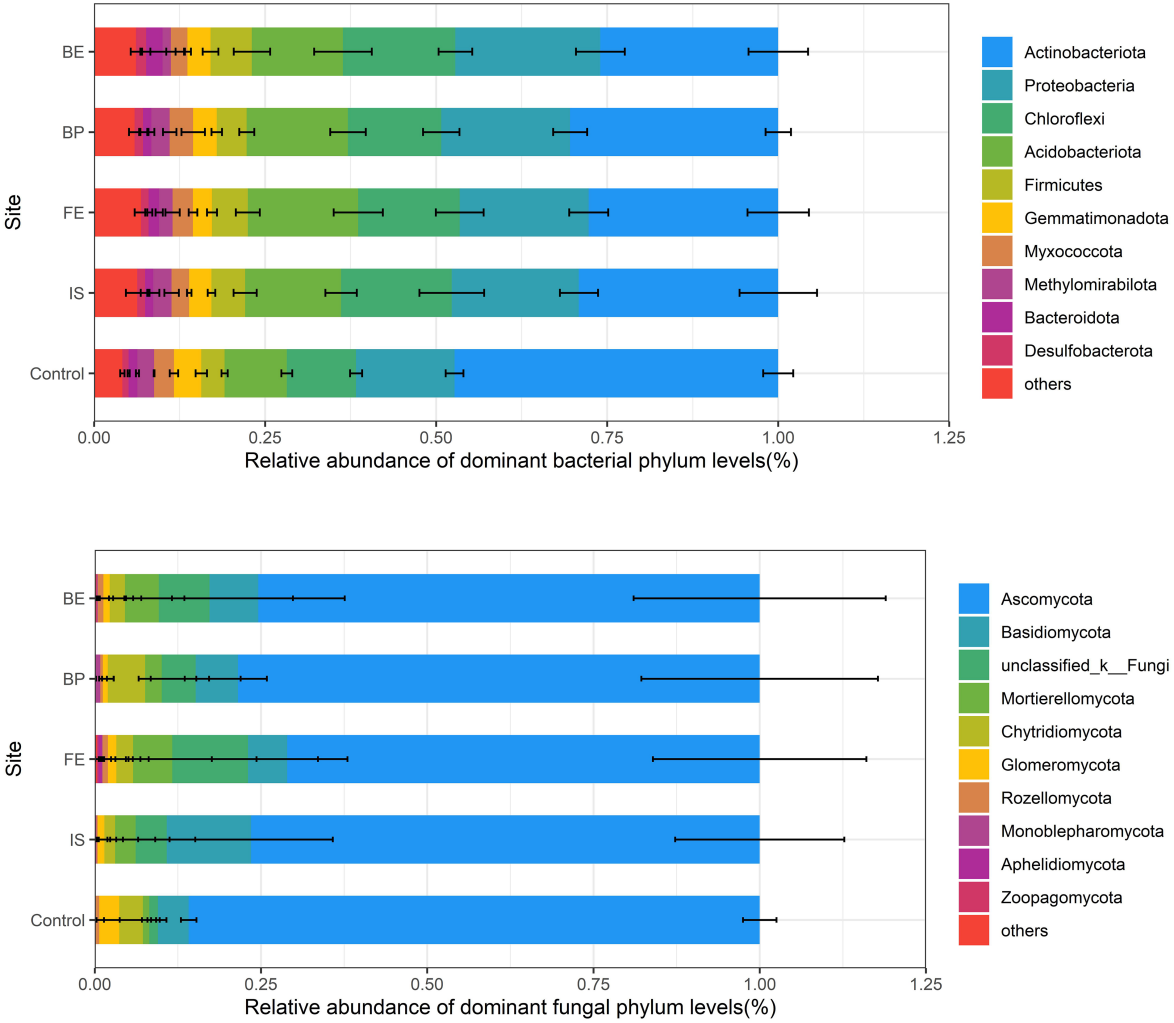
Relative abundance of dominant phyla of soil bacteria and fungi at different sites of PV panels.

At the phylum level, the relative abundance of the top 10 dominant bacteria in all sites was higher than 91% (29.77% Actinobacteriota, 18.98% Proteobacteria, 14.88% Chloroflexi and 14.15% Acidobacteriota, 5.01% Firmicutes, 3.27% Gemmatimonadota, 2.86% Myxococcota, 2.15% Methylomirabilota, 1.56% Bacteroidota and 1.23% Desulfobacterota). PV panels had significant effects on the relative abundance of Actinobacteriota, Proteobacteria, Acidobacteriota and Methylomirabilota (*p* < 0.05), decreased the relative abundance of Actinobacteriota, while increased the relative abundance of Proteobacteria, Acidobacteriota, and Methylomirabilota. There was no significant difference in the relative abundance of Actinobacteriota among different sites of PV panels. The relative abundance of Proteobacteria in BE was the highest, Actinobacteriota in FE was the highest, Methylomirabilota in IS and BP were significantly higher than that of FE and BE (Figure 3; Supplementary Table S1). In addition, LEfSe analysis (threshold of 3) showed that there were differences in bacterial community composition between Control and different sites of PV panels. Specifically, Proteobacteria, Actinobacteriota, Gemmatimonadota, Bacteroidota and Patescibacteria were the most enriched in the Control. Chloroflexi, Gemmatimonadota, Cyanobacteria, Actinobacteriota and Methylomirabilota in IS. Unclassified_k__norank_d__Bacteria, Verrucomicrobiota, Bacteroidota, Actinobacteriota, Planctomycetota, Proteobacteria, Myxococcota and Proteobacteria in FE. Methylomirabilota in BP. Proteobacteria and Actinobacteriota in BE (Supplementary Figure S5).

At the phylum level, the top 10 dominant fungi with relative abundance in all sites accounted for more than 99% (76.16% Ascomycota, 7.80% Basidiomycota, 6.75% Unclassified_k__Fungi, 3.88% Mortierellomycota, 3.06% Chytridiomycota, 1.15% Glomeromycota, 0.56% Rozellomycota, 0.27% Monoblepharomycota, 0.12% Aphelidiomycota and 0.07% Zoopagomycota). PV panels had no significant effect on the relative abundance of fungal dominant phyla, and there was no significant difference between the relative abundance of dominant phyla at different sites of PV panels and Control (Figure 3; Supplementary Table S2). LEfSe analysis showed that Basidiomycota, Ascomycota, Mucoromycota and Glomeromycota were the most enriched in Control. Ascomycota in IS. Basidiomycota, Zoopagomycota, Ascomycota and Aphelidiomycota in FE. Ascomycota and Glomeromycota in BP, BE is Ascomycota (Supplementary Figure S6).

### Relationships between biotic and abiotic properties and microbial communities

**Figure 4:**
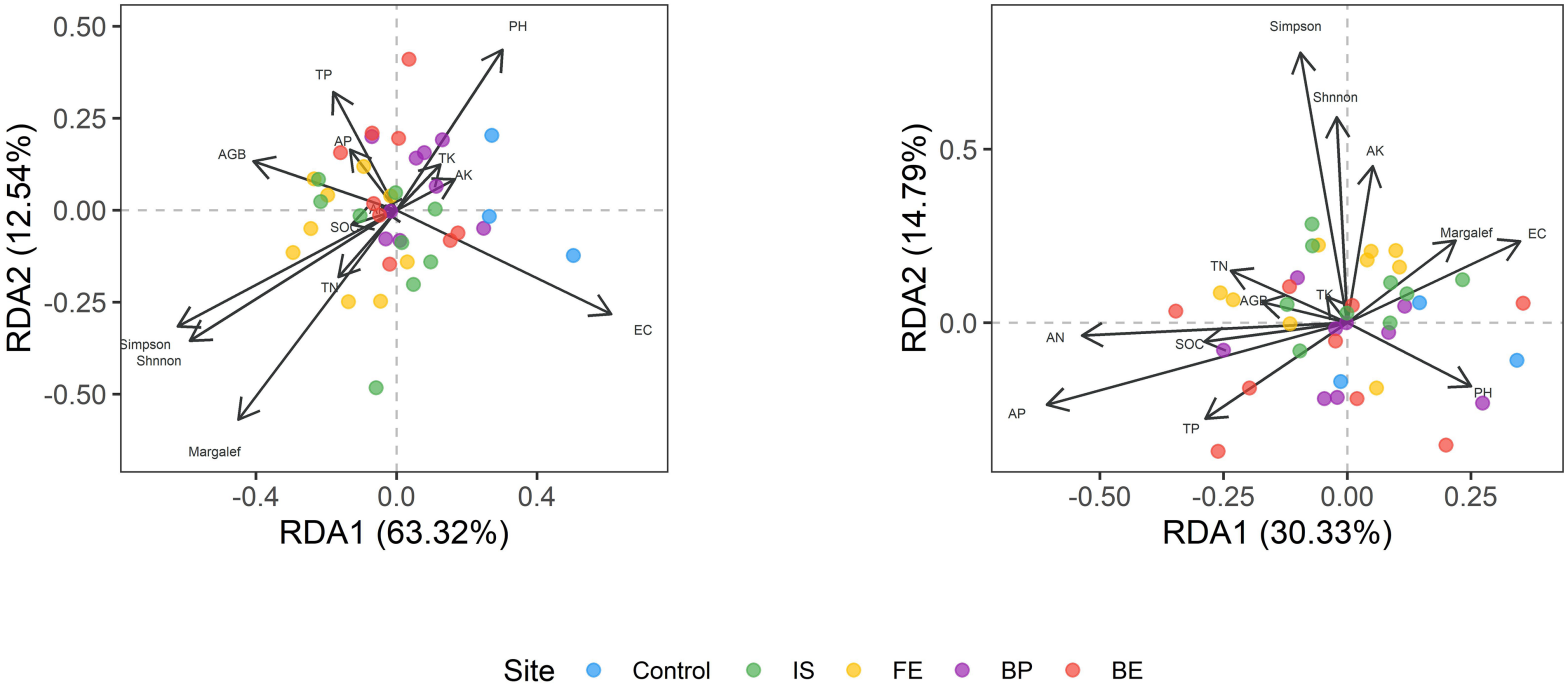
RDA analysis of soil bacteria and fungi with plant communities and soil properties at different sites of PV panels.

**Figure 5:**
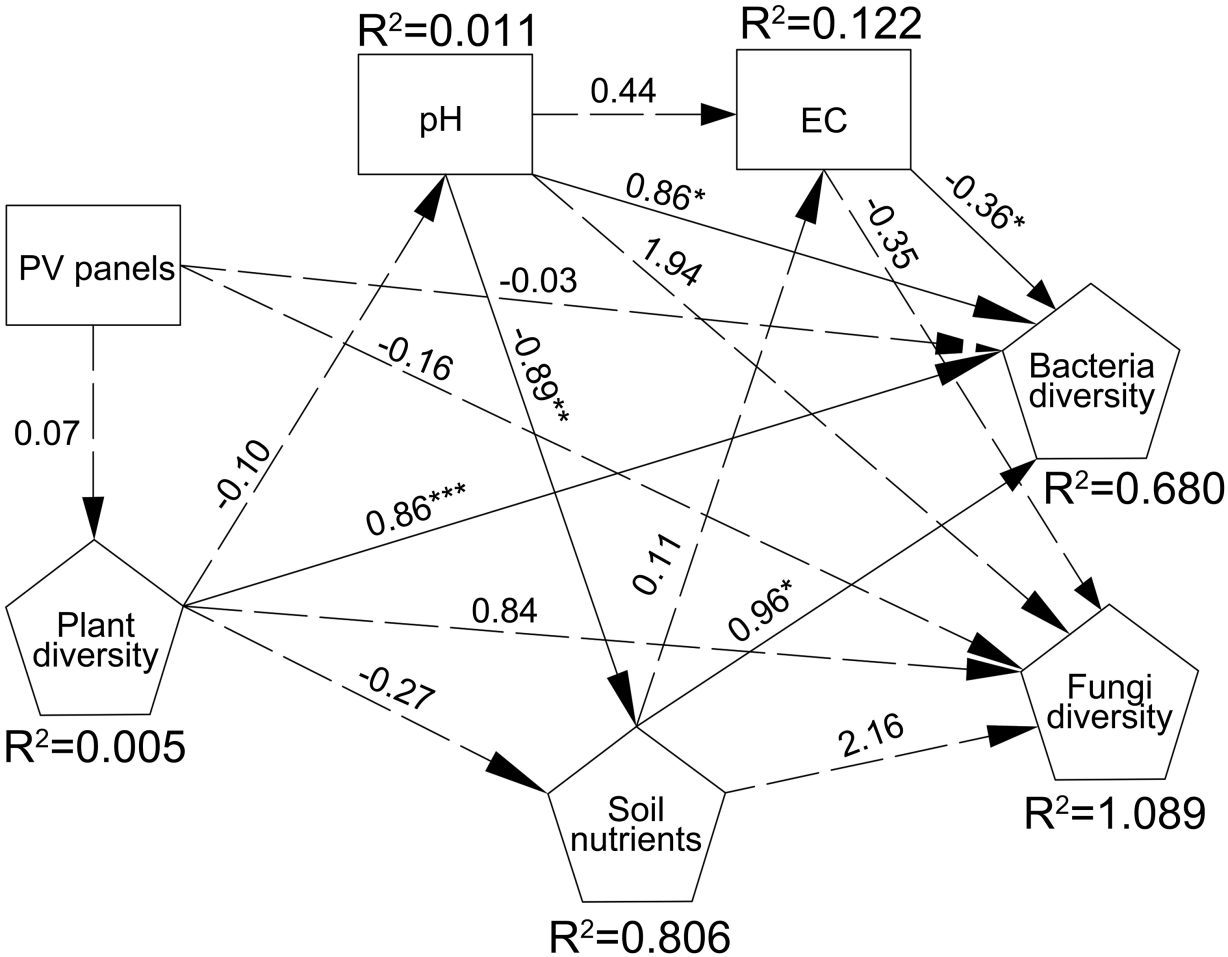
SEM model of the interaction between plant communities, soil properties and soil microorganisms (p = 0.003, χ^2^/df = 1.036, GFI =0.809, RMSEA = 0.031).

RDA analysis was performed to illustrate the relationship between bacterial and fungal community composition and soil properties. The first two RDA1 and RDA1 axes explained 61.62 and 12.82% of the variation in overall bacterial community composition, respectively. RDA results showed that EC, Margale and total AGB affected the composition of bacterial community (*p* = 0.004; *p* = 0.035; *p* = 0.025) (Figure 4; Supplementary Table S3). The first two RDA1 and RDA1 axes explained 29.11 and 14.59% of the variation in fungal community composition, respectively. RDA results showed that AN and AP significantly affected the composition of fungal communities (*p* = 0.034; *p* = 0.018) (Figure 4; Supplementary Table S3). SEM model was used to describe the effects of PV panels on plant diversity and soil microorganisms, and the interactions among plant diversity, soil properties and soil microorganisms. SEM model was used to describe the interaction between plant communities, soil properties and soil microorganisms (*p* = 0.003, χ^2^/df = 1.036, GFI =0.809, RMSEA = 0.031). SEM results showed that plant diversity directly affected soil bacterial diversity or indirectly affected soil bacterial diversity by affecting soil pH (*p* < 0.05) (Figure 5).

**Figure 6:**
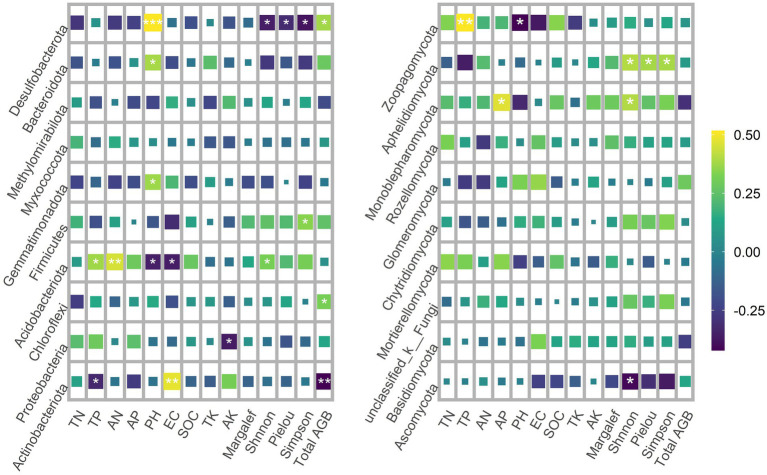
Spearman rank correlation analysis of dominant phyla of soil bacteria and fungi with plant diversity and soil properties.

Spearman rank correlation analysis showed that Actinobacteriota was negatively correlated with TP and total AGB, but positively correlated with EC. Proteobacteria was negatively correlated with AK. Chloroflexi was positively correlated with total AGB. Acidobacteriota was negatively correlated with pH and EC, and positively correlated with TP, AN and Shnnon-Wiener. Firmicutes was positively correlated with Simpson diversity. Gemmatimonadota was positively correlated with pH. Bacteroidota was positively correlated with pH. Desulfobacterota is negatively correlated with pH and the positive correlation total AGB, and negatively correlated with Shnnon-Wiener and Simpson diversity (Figure 6).

In the dominant phylum of fungi, Ascomycota was negatively correlated with Shnnon-Wiener. Monoblepharomycota was positively correlated with AP and Shnnon-Wiener. Aphelidiomycota was positively correlated with Shnnon-Wiener and Simpson diversity. Zoopagomycota was negatively correlated with pH and positively correlated with TP (Figure 6).

## Discussion

### Effects of PV panels on vegetation communities and soil properties

Temperature and precipitation were the main moderators of grassland community composition and richness (Yang et al., 2011; Jonas et al., 2015). PV panels significantly increased the diversity of plant communities for the following reasons: on the one hand, grasses have shallow and fibrous roots, usually distributed in the soil surface (Mackie et al., 2019), while forbs and sedges have deep roots and are resource conservative (Yang et al., 2011). The PV panels reduced water collection and lower potential evaporation, which promoted soil water retention and improved the effective water content of deep soil. By affecting interspecific competition, it inhibited the growth of dominant PG and created ecological niches, and to a certain extent promoted the growth of subdominant PF, PS and ABH. On the other hand, numerous studies have demonstrated that temperature changes have different effects on different plant groups of PF, PS and PG (Peng et al., 2017; Ganjurjav et al., 2018). The absorption of solar radiation by PV panels effectively reduced the soil temperature, and the change of temperature effectively regulated the interspecific relationship and promoted the growth of different species. In addition, the adaptation or plasticity to shading varies greatly among plants (Semchenko et al., 2011). Faster growing and larger hybrid grasses have a disproportionate advantage in increasing their light capture capacity during shading through an increase in stem and leaf elongation and specific leaf area (SLA) (Ballare et al., 1994; Schwinning and Weiner, 1998) and resulted in competitive exclusion of smaller or slower-growing grass individuals (Hautier et al., 2009). In conclusion, PV panels effectively inhibited the growth of dominant PG by changing abiotic factors, promoted the growth of subdominant species, and then increased the diversity of grassland plant communities, and changed the composition and structure of plant communities.

Water availability in semi-arid regions limits plant growth and ecosystem productivity (Niu et al., 2008). Precipitation from January to July was reported to be the main cause of community biomass fluctuation in the same area (Bai et al., 2004). PV panels increased soil available water content, which not only directly promoted community photosynthesis and plant growth (Bai et al., 2010; Li et al., 2016), and indirectly increased aboveground biomass by increasing plant community diversity (Isbell et al., 2013). Numerous studies have demonstrated a positive correlation between biodiversity and biomass production (Isbell et al., 2013; Hautier et al., 2014; Hautier and Van der Plas, 2022). However, there were significant differences in total AGB among different sites of PV panels, and the higher soil water content at FE resulted in the most significant increase in total AGB. Total AGB under BP was lower than that at other sites of the PV panels, which may be attributed to, on the one hand, the shading of the PV panels reduced precipitation; on the other hand, the low light level and thick litter layer under BP inhibited plant growth (Seitz et al., 2016). It is worth noting that from the perspective of homogeneity, IS was least affected by PV panels in different sites under PV panels, compared with IS, the plant species diversity and total AGB of FE were significantly improved, and BP were significantly reduced, which may be that the PV panels were oblique arrangement, so that the soil moisture content of FE was significantly higher than other sites under the PV panels, and BP to precipitation and light inhibited the growth of plants.

Plants and soil are inseparable (Pugnaire et al., 2019), plant species replacement caused by climate change will change material input and decomposition by changing the form of plant litter production and root deposition, thus affecting soil properties (Bardgett et al., 2013: Davidson and Janssens, 2006; Kulmatiski et al., 2008; Glassman et al., 2018). However, our results show that changes in plant community composition did not significantly alter soil properties. The study of Lambert et al. (2021) also showed that the construction of PV panels did not reduce the C and N contents, nor did it reduce the soil chemical properties. The reasons may be that the PV panel laying time in the study site is short, and the soil properties have no obvious difference with the Control due to the hysteresis of the response to plant community changes. In addition, low light levels increased litter production (Seitz et al., 2016), and increased soil material input, resulting in slightly higher soil nutrient content at BP than at other sites under PV panels.

### Effects of PV panels on soil microbial diversity and structure

Soil moisture strongly influenced microbial diversity (Orwin et al., 2015; Yang et al., 2019). Different sites under PV panels (especially FE) significantly increased soil available water content, and increased soil water availability promoted microbial growth. PV panels significantly increased the richness and diversity of soil bacterial communities, changed the composition of bacterial communities, and increased the richness of fungal communities. FE bacterial diversity increased significantly compared to IS, while BP decreased significantly, possibly due to the redistribution of precipitation by PV panels. NMDS analysis showed that the composition of bacterial and fungal communities of PV panels was similar to that of controls, which may be due to the delayed response of bacteria and fungi due to the short PV placement time.

Actinobacteriota and Proteobacteria have also been identified as the major bacterial communities in soils (Banerjee et al., 2018). In this study, Actinobacteriota and Proteobacteria were the most dominant phyla in all sites. Consistent with previous studies, Actinobacteriota plays a key role in desert grassland soils, followed by Proteobacteria, a more important phylum in typical grassland soils (Wang et al., 2017). It has been reported that Actinobacteriota is a group of eutrophic bacteria whose relative abundance increases with the size of soil C and N pools (Fierer et al., 2007; Li et al., 2014). Acidobacteria is an oligotrophic bacterium with poor tolerance and low temperature tolerance (Fierer et al., 2007; Yao et al., 2017). The relative abundance of Actinobacteriota at different sites under PV panels decreased significantly, while Acidobacteria increased significantly, possibly because the laying of PV panels reduced soil nutrition. The relative abundance of Proteobacteria increased significantly under the artificial interference of PV panels. Xiao et al. (2021) and Kolton et al. (2011) also reported that the soil disturbed by mining was significantly enriched in Proteobacteria. Proteobacteria is a fast-growing bacterium that can utilize a variety of carbon sources (i.e., syntrophic bacteria) (Yao et al., 2017). This may be related to the extensive biodegradative and metabolic properties of Proteobacteria bacteria and their ability to inhabit a variety of habitats (Sinkko et al., 2013). Proteobacteria may grow rapidly when available substrates are unstable (e.g., soil disturbance) (Zhang et al., 2016). LefSe analysis showed that there were significant differences in dominant phyla at different sites under PV panels, and the significantly different species in FE were significantly higher than those in other sites, which indicated that PV panels, especially FE, significantly changed the diversity and structure of soil bacteria.

The major fungal phyla in soils worldwide are Ascomycota and Basidiomycota (Tedersoo et al., 2014). This is consistent with our results that Ascomycota was the dominant phylum in different fungal communities, followed by Basidiomycota. Ascomycotion is an oligotrophic fungus that can tolerate harsh conditions (Chen et al., 2017), Basidiomycota is a eutrophic bacterium (Sterkenburg et al., 2015), The lack of resources may be the reason why the relative abundance of Ascomycota and Basidiomycota did not change under different sites of PV panels.

### Biotic and abiotic characteristics in relation to microbial communities

Changes in precipitation and temperature affect soil microbial communities directly by altering their growth and activity (Bardgett et al., 2008) and also by altering their function on individual plants (e.g., root biomass production) (Orwin et al., 2010), or plant community composition indirectly affects soil microbial populations (Kardol et al., 2007; Luo et al., 2017; Ren et al., 2018). RDA, Spearman rank correlation and SEM showed that plant diversity was significantly correlated with the diversity and composition of bacterial and fungal communities. Plant diversity directly affected soil bacterial diversity or indirectly affected soil bacterial diversity by affecting soil pH. PV panels alter plant community composition, which changes the quality and/or quantity of root exudates and litter entering the soil, which in turn affects microbial community composition and activity (Bardgett and Wardle, 2010). Studies have shown that low-quality, refractory, slow-growing species determine fungus-dominated microbial community composition, while nutrient-rich, fast-growing species (such as miscellaneous grasses) determine bacteria-dominated microbial community composition (Orwin et al., 2010; de Vries et al., 2012; Pugnaire et al., 2019). PV significantly increased fast-growing species such as PF, which provided a good growth substrate for bacteria and drove the growth of bacterial communities. In addition, each plant has unique root exudate characteristics, and a richer plant community may have created a niche for the growth of different microorganisms (Zak et al., 2003; Bartelt-Ryser et al., 2005; Loranger-Merciris et al., 2006). Meanwhile, bacterial diversity and dominant phyla were significantly correlated with total AGB, which was consistent with the conclusion of Bardgett and McAlister (1999), suggesting that productivity may be a mechanism linking plant species richness and soil community structure.

Salinity (EC) was a major factor affecting bacterial community composition (Lozupone and Knight, 2007), which was consistent with our RDA results. Spearman rank correlation analysis showed that Actinobacteriota and Acidobacteriota were significantly correlated with EC. Previous studies have shown that Firmicutes, Bacteroidota and Proteobacteria have high salt tolerance, and their relative abundance is related to EC (Antunes et al., 2011; Geyer et al., 2014). With the increase of soil salinity, only a limited number of bacterial groups can withstand the enormous pressure of high salinity, resulting in changes in the relative abundance of microorganisms and community diversity (Rath et al., 2018). In addition, salinity is often correlated with OM inputs and soil pH (Wong et al., 2008; Rengasamy, 2010). SEM results showed that bacterial diversity was affected by soil pH. The correlation analysis shows that Desulfobacterota is positively correlated with pH. Since most bacterial taxa have relatively narrow growth tolerances, changes in soil pH may directly affect changes in bacterial communities (Lauber et al., 2008; Rousk et al., 2010). Proteobacteria was negatively correlated with AK. K is an essential element that regulates cellular pH in microorganisms and drives uptake of soil nutrients by regulating cellular osmotic pressure (Pan et al., 2020). Soil bacteria have been reported to influence K solubility and availability, which in turn influences the selection of specific bacteria associated with K concentrations (Pereira et al., 2014; Emmert et al., 2021). There is no general consensus on the effect of P on microbial community composition, as P has negative, neutral, or positive effects on soil microorganisms in terrestrial ecosystems (DeForest et al., 2012; Liu et al., 2012; Huang et al., 2016). RDA showed that the fungal community composition was affected by AP content. Correlation analysis showed that the relative abundance of Actinobacteriota, Acidobacteriota, Monoblepharomycota, and Zoopagomycota were significantly correlated with P content. This may be due to the decrease in soil pH by P application, which drives the change in the relative abundance of different bacteria (Jones et al., 2009). In addition, AN also significantly affected fungal community composition. Studies have shown that soil N content can directly affect soil microorganisms, or indirectly affect soil microorganisms by changing soil C availability, C/N ratio and soil pH (Treseder, 2008; Serna-Chavez et al., 2013). Taken together, our study provides further evidence that PV panels drive soil microbial changes by influencing abiotic and biotic conditions, and that soil (microenvironment) heterogeneity is a factor that maintains soil microbial community diversity.

## Conclusion

In conclusion, our study found that PV panels significantly increased grassland plant community diversity by driving microclimate change. FE increased precipitation accumulation and plant diversity directly and indirectly changed the diversity of soil bacterial and fungal communities. PV panels decreased the relative abundance of Actinobacteriota, while increased that of Proteobacteria, Acidobacteriota, and Methylomirabilota. EC, Margalef’s richness and total AGB were the main factors affecting the composition of bacterial community, while AN and AP were the main factors affecting the composition of fungal community. Different microorganisms were affected by different soil properties, and the increase of plant diversity driven by abiotic factors was the main factor maintaining the diversity of soil microbial community and ecosystem function. The results provided important information for further understanding the effect of photovoltaic panel laying on grassland ecosystem function, and were of great significance for maintaining grassland ecosystem function.

## Data availability statement

The datasets presented in this study can be found in online repositories. The names of the repository/repositories and accession number(s) can be found at: Sequence Read Archive (SRA) of the NCBI database under the BioProjectID PRJNA895197.

## Author contributions

ZyB, ZjB, and MW conceived and designed this study. ZyB drafted the original manuscript. MW provided very constructive suggestions for revisions. ZyB, MW, ZjB, SQ, MZ, LK, RS, and MW contributed to the sampling and data analysis. All authors read and approved the final manuscript.

## Funding

This project was supported by the new energy field in Daqing of China “The open competition mechanism to select the best candidates” scientific and technological project (2021BD05) and the Special Fund Project for Returnees Studying Abroad of Heilongjiang Province (2021294).

## Conflict of interest

The authors declare that the research was conducted in the absence of any commercial or financial relationships that could be construed as a potential conflict of interest.

## Publisher’s note

All claims expressed in this article are solely those of the authors and do not necessarily represent those of their affiliated organizations, or those of the publisher, the editors and the reviewers. Any product that may be evaluated in this article, or claim that may be made by its manufacturer, is not guaranteed or endorsed by the publisher.
