# Supplementary figures and images for: Photovoltaic panels have altered grassland plant biodiversity and soil microbial diversity
Source: Front Microbiol. 2022 Dec 15;13:1065899. doi: 10.3389/fmicb.2022.1065899 (PMC9797687; doi:10.3389/fmicb.2022.1065899)

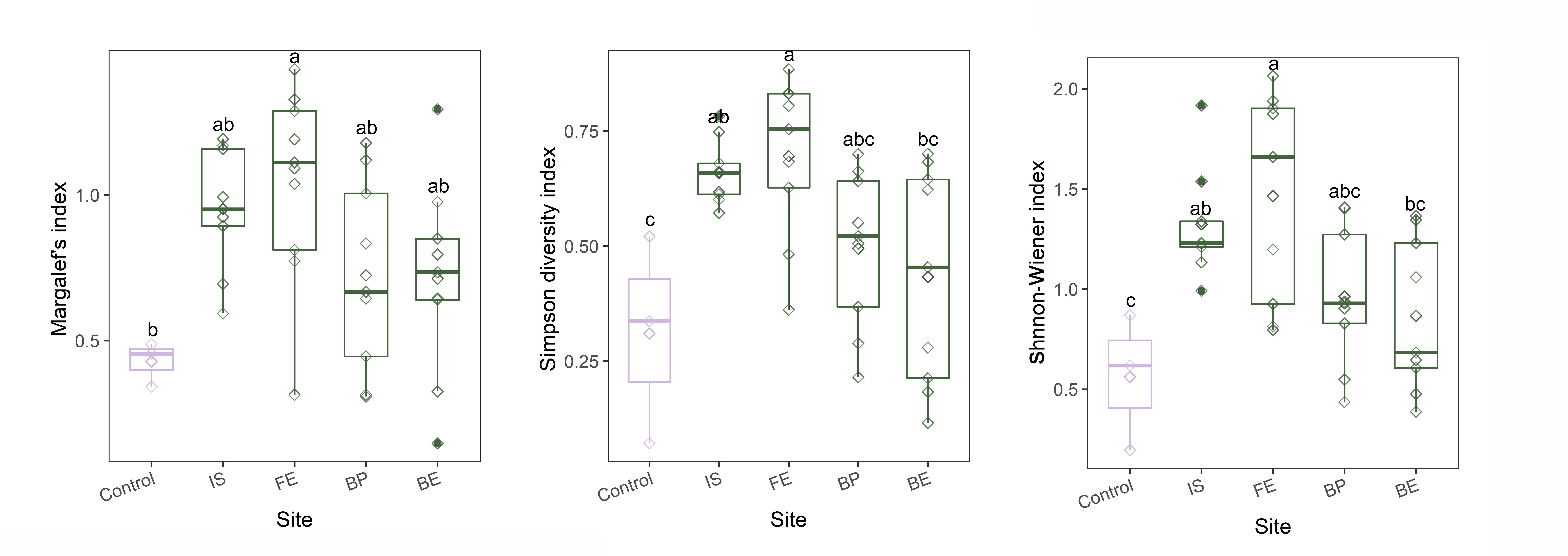

Supplement: Supplementary file 1 [file Image_1.jpg]

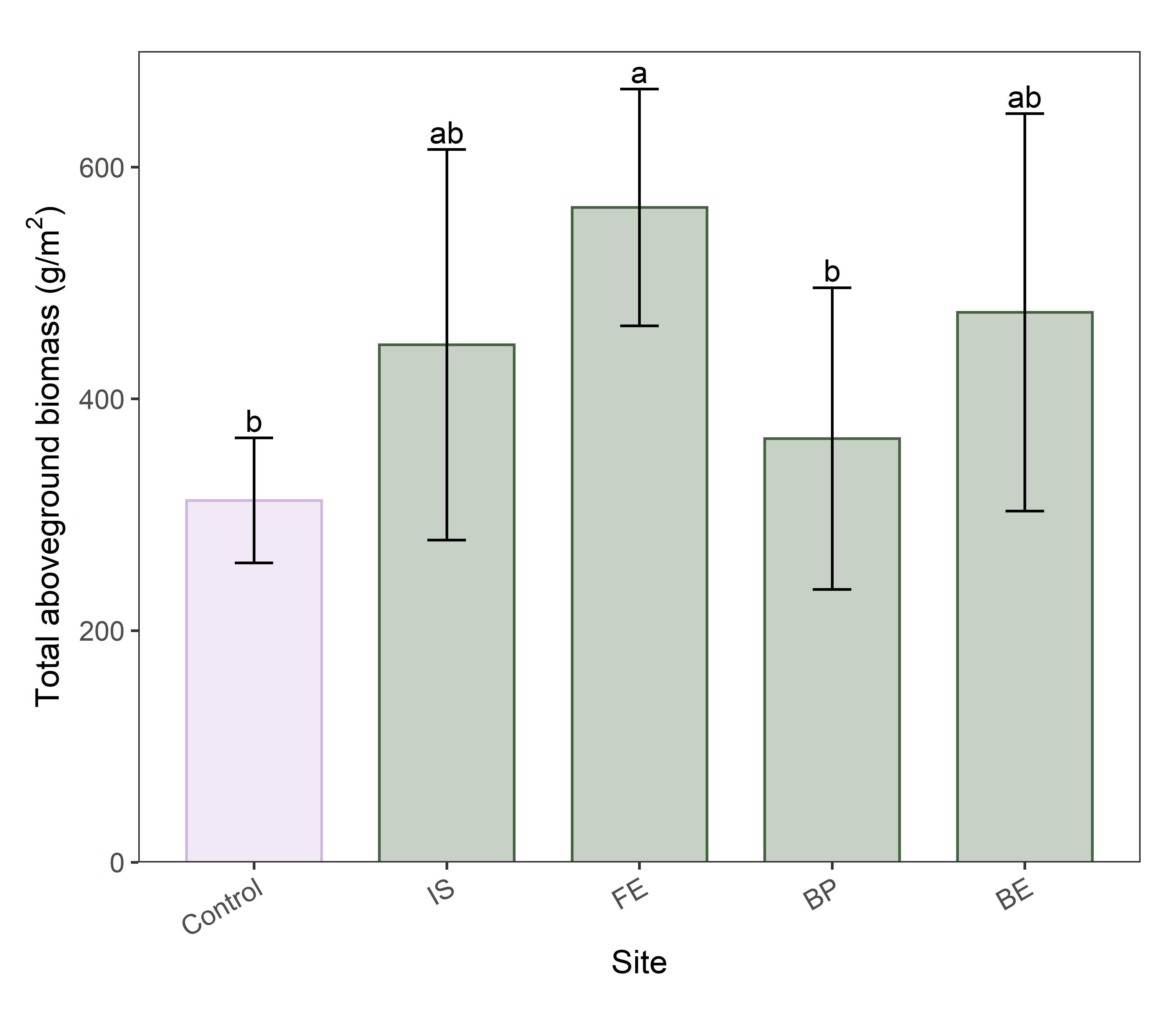

Supplement: Supplementary file 2 [file Image_2.jpeg]

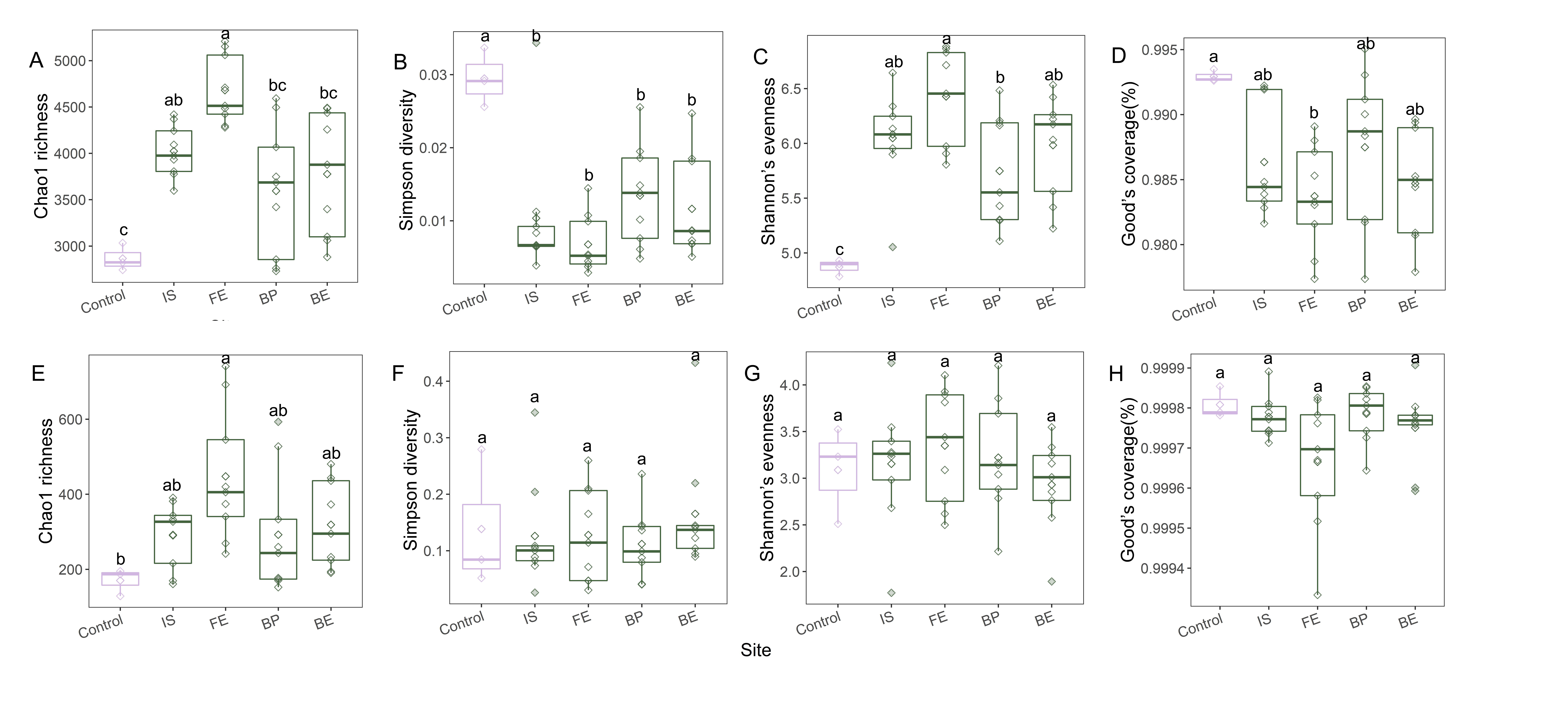

Supplement: Supplementary file 3 [file Image_3.jpg]

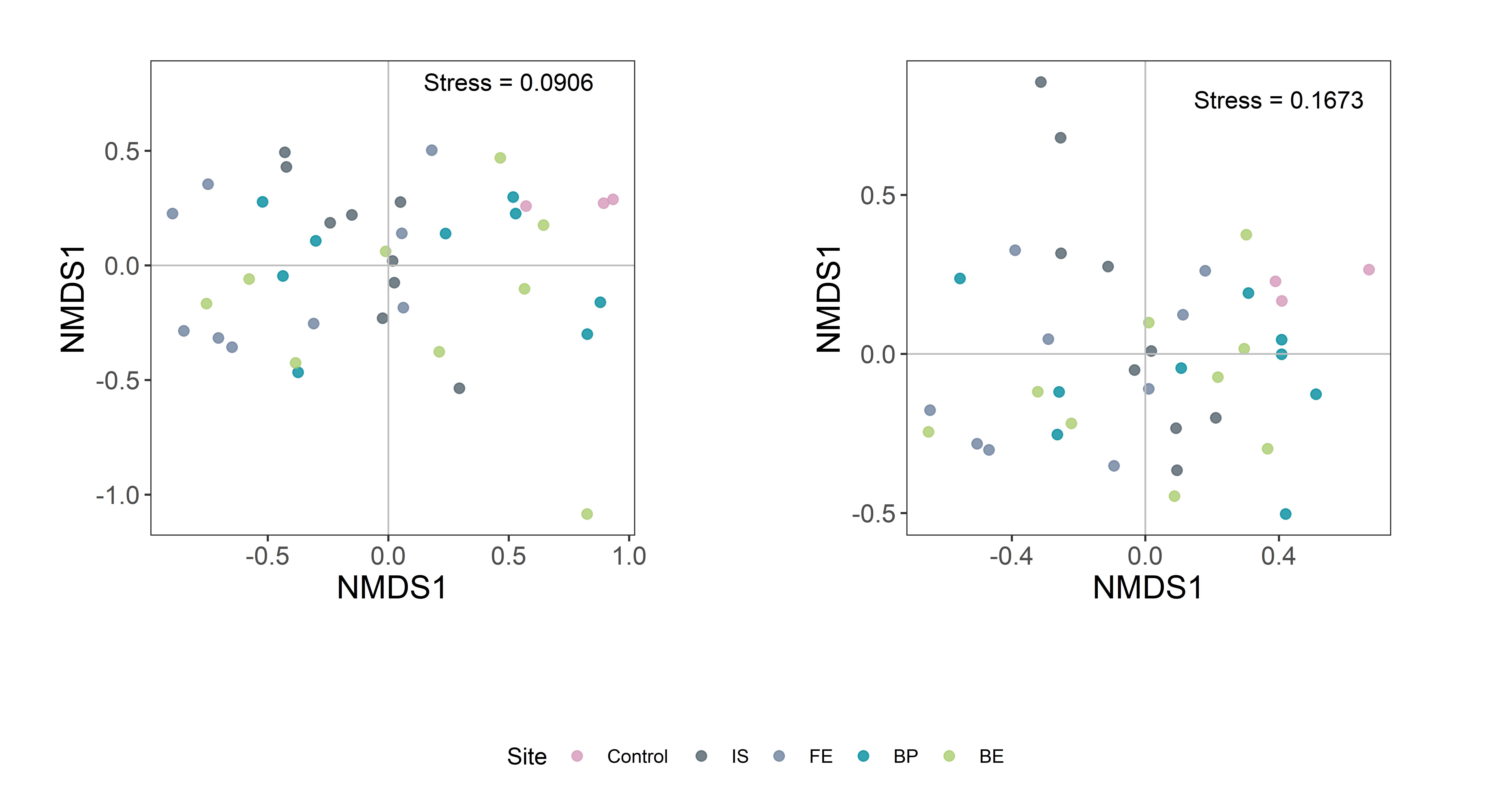

Supplement: Supplementary file 4 [file Image_4.jpeg]

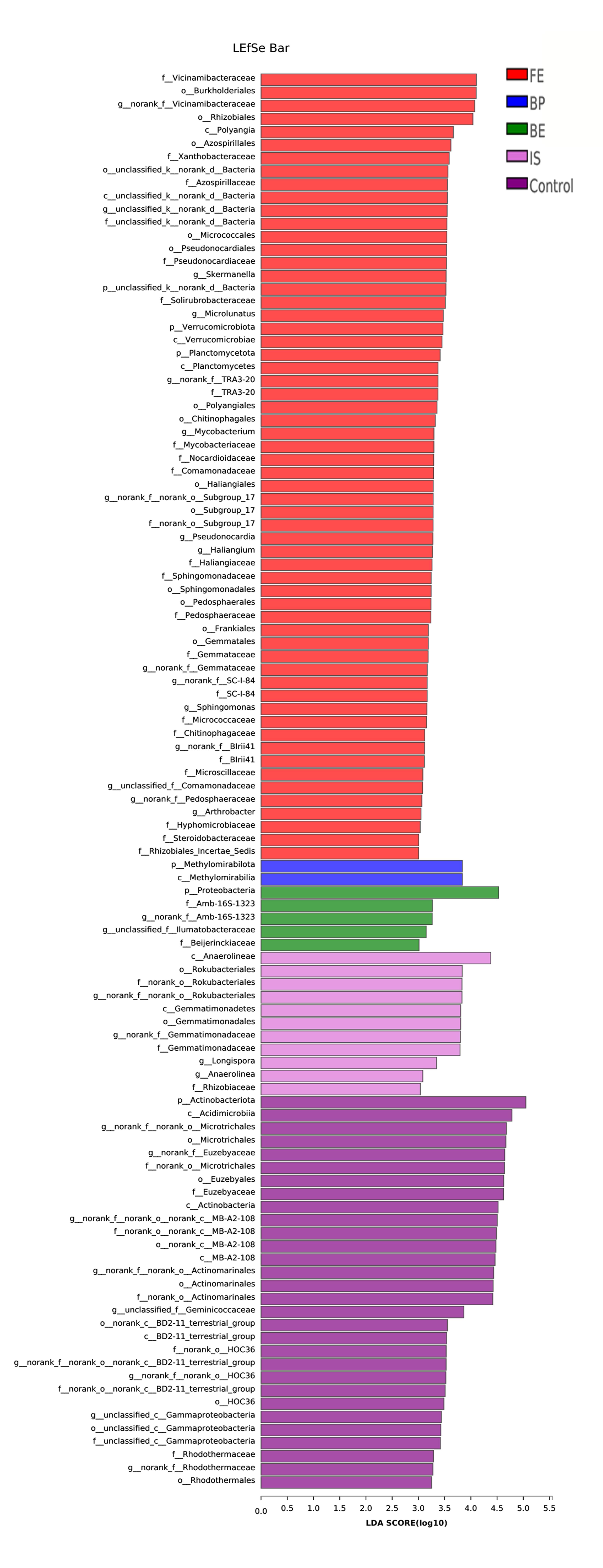

Supplement: Supplementary file 5 [file Image_5.jpg]

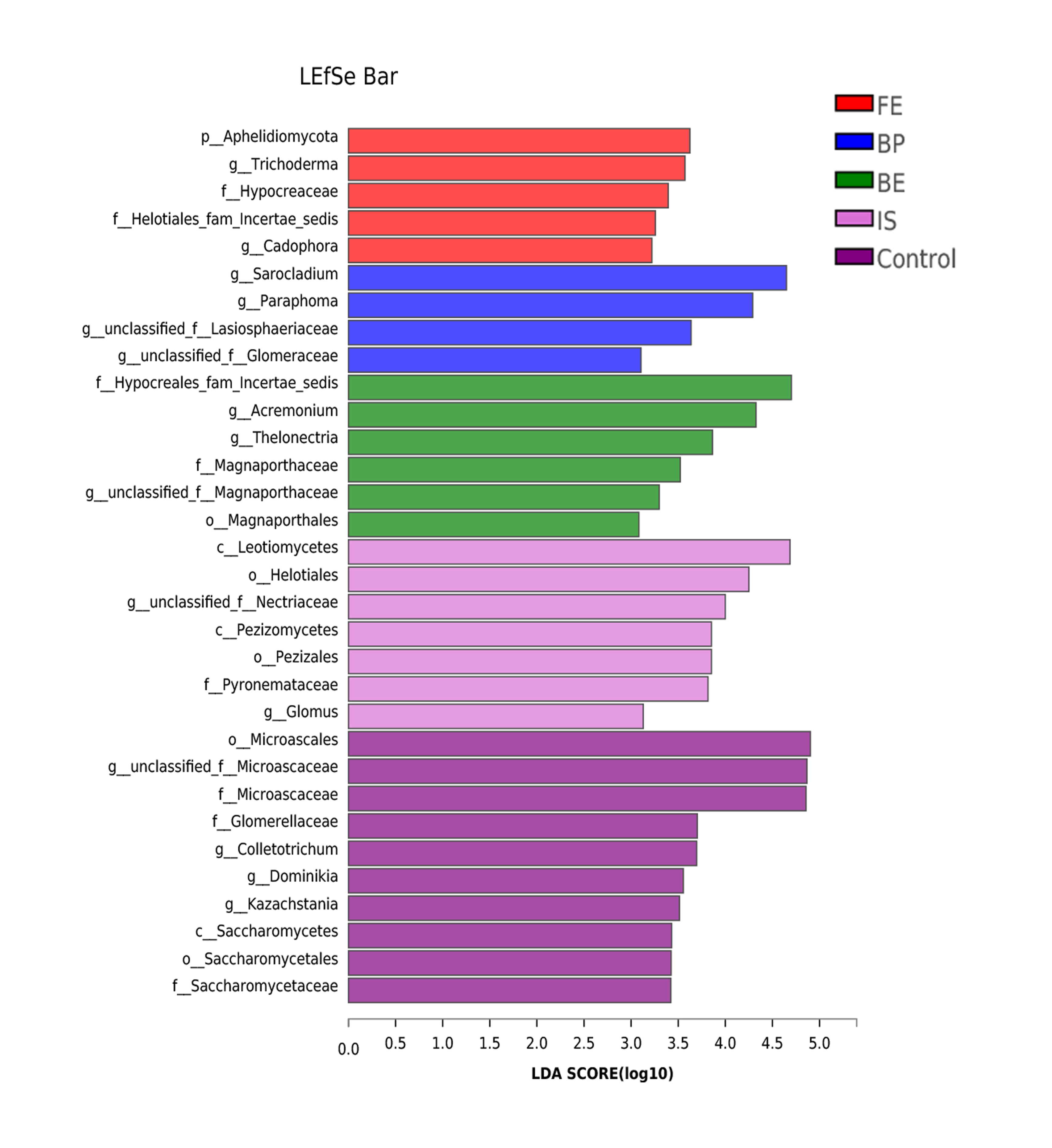

Supplement: Supplementary file 6 [file Image_6.jpg]
