# Supplementary material for: Photovoltaic panels have altered grassland plant biodiversity and soil microbial diversity
Source: Front Microbiol. 2022 Dec 15;13:1065899. doi: 10.3389/fmicb.2022.1065899 (PMC9797687; doi:10.3389/fmicb.2022.1065899)
Supplement: Supplementary file 7 [file Table_1.docx]

Table S1 One-way ANOVA analysis of the effect of PV panels on bacterial dominant phyla.

| Site | Actinobacteriota | Proteobacteria | Chloroflexi | Acidobacteriota | Firmicutes | Gemmatimonadota | Myxococcota | Methylomirabilota | Bacteroidota | Desulfobacterota | others |
| --- | --- | --- | --- | --- | --- | --- | --- | --- | --- | --- | --- |
| Control | 0.47±0.03a | 0.14±0.02b | 0.10±0.01a | 0.09±0.01b | 0.03±0.01a | 0.04±0.01a | 0.03±0.01a | 0.02±0.00ab | 0.01±0.00a | 0.01±0.00a | 0.04±0.00b |
| IS | 0.29±0.06b | 0.19±0.03ab | 0.16±0.05a | 0.14±0.02ab | 0.05±0.02a | 0.03±0.01a | 0.03±0.00a | 0.03±0.01a | 0.01±0.01a | 0.01±0.01a | 0.06±0.01ab |
| FE | 0.28±0.05b | 0.19±0.03ab | 0.15±0.04a | 0.16±0.04a | 0.05±0.02a | 0.03±0.01a | 0.03±0.01a | 0.02±0.01ab | 0.02±0.01a | 0.01±0.01a | 0.07±0.01a |
| BP | 0.30±0.02b | 0.19±0.03ab | 0.14±0.03a | 0.15±0.03ab | 0.04±0.01a | 0.03±0.01a | 0.03±0.02a | 0.03±0.01a | 0.01±0.00a | 0.01±0.01a | 0.06±0.01ab |
| BE | 0.26±0.05b | 0.21±0.04a | 0.16±0.03a | 0.13±0.04ab | 0.06±0.03a | 0.03±0.01a | 0.02±0.01a | 0.01±0.01b | 0.02±0.03a | 0.01±0.01a | 0.06±0.01ab |
| *F* | 13.590 | 2.900 | 2.346 | 2.705 | 1.419 | 1.308 | 1.377 | 3.498 | 0.703 | 0.706 | 3.315 |
| *P* | < 0.001 | 0.036 | 0.0742 | 0.046 | 0.249 | 0.287 | 0.263 | 0.017 | 0.595 | 0.593 | 0.021 |

Table S2 One-way ANOVA analysis of the effect of PV panels on fungi dominant phyla.

| Site | Ascomycota | Basidiomycota | unclassified_k__Fungi | Mortierellomycota | Chytridiomycota | Glomeromycota | Rozellomycota | Monoblepharomycota | Aphelidiomycota | Zoopagomycota | others |
| --- | --- | --- | --- | --- | --- | --- | --- | --- | --- | --- | --- |
| Control | 0.86±0.03a | 0.05±0.01a | 0.01±0.00a | 0.01±0.00a | 0.04±0.04a | 0.03±0.04a | 0.01±0.01a | 0.00±0.00a | 0.00±0.00a | 0.00±0.00a | 0.00±0.00a |
| IS | 0.76±0.13a | 0.13±0.13a | 0.05±0.05a | 0.03±0.03a | 0.02±0.01a | 0.01±0.01a | 0.00±0.00a | 0.00±0.00a | 0.00±0.00a | 0.00±0.00a | 0.00±0.00a |
| FE | 0.71±0.17a | 0.06±0.05a | 0.11±0.16a | 0.06±0.06a | 0.03±0.01a | 0.01±0.02a | 0.01±0.01a | 0.00±0.00a | 0.00±0.00a | 0.00±0.00a | 0.00±0.00a |
| BP | 0.78±0.19a | 0.06±0.05a | 0.05±0.07a | 0.02±0.04a | 0.06±0.08a | 0.01±0.01a | 0.00±0.01a | 0.01±0.02a | 0.00±0.00a | 0.00±0.00a | 0.00±0.00a |
| BE | 0.75±0.20a | 0.07±0.13a | 0.08±0.13a | 0.05±0.04a | 0.02±0.03a | 0.01±0.02a | 0.01±0.02a | 0.00±0.00a | 0.00±0.00a | 0.00±0.00a | 0.00±0.00a |
| *F* | 0.491 | 0.786 | 0.751 | 1.309 | 1.131 | 0.862 | 0.758 | 0.609 | 0.804 | 0.888 | 2.546 |
| *P* | 0.742 | 0.542 | 0.564 | 0.286 | 0.358 | 0.496 | 0.560h | 0.659 | 0.531 | 0.481 | 0.057 |

Table S3 RDA analysis of soil bacteria and fungi with plant communities and soil properties at different sites of PV panels.

|  | bacterial | | fungi | |
| --- | --- | --- | --- | --- |
|  | F | *P* | F | *P* |
| TN | 0.71 | 0.595 | 0.76 | 0.727 |
| AN | 0.42 | 0.861 | 1.75 | 0.041 |
| TP | 1.19 | 0.293 | 1.18 | 0.243 |
| AP | 0.52 | 0.711 | 1.93 | 0.023 |
| TK | 0.42 | 0.835 | 0.74 | 0.757 |
| AK | 0.81 | 0.490 | 1.06 | 0.369 |
| SOC | 0.49 | 0.745 | 0.77 | 0.702 |
| pH | 1.67 | 0.143 | 0.83 | 0.645 |
| EC | 4.76 | 0.006 | 1.18 | 0.259 |
| Margalef’ s richness | 2.60 | 0.050 | 0.87 | 0.597 |
| Shnnon-Wiener | 2.40 | 0.064 | 1.27 | 0.218 |
| Simpson diversity | 0.63 | 0.612 | 1.19 | 0.287 |
| Total AGB | 2.85 | 0.034 | 0.81 | 0.674 |
